# Do job demands and resources generalize across academic career stages? Implications for burnout and work engagement

**DOI:** 10.3389/ijph.2026.1610023

**Published:** 2026-10-12

**Authors:** May Thet Nu Noe, Pascal Wild, Jana Lasser, Irina Guseva Canu

**Affiliations:** 1 Centre for Primary Care and Public Health (Unisanté), University of Lausanne, Lausanne, Switzerland; 2 Interdisciplinary Center IDea_Lab, University of Graz, Graz, Austria

**Keywords:** academic researchers, burnout, JD-R framework, structural equation modelling, work engagement

## Abstract

**Objectives:**

The Job Demands-Resources (JD-R) framework is widely used to explain burnout and work engagement, but its applicability across academic career stages remains unclear. This study examined whether the JD-R framework generalizes across junior and senior researchers.

**Methods:**

This cross-sectional survey collected data from 3,101 researchers across 27 European countries, measuring burnout, work engagement, four job demands (illegitimate tasks, job insecurity, interpersonal conflict, work-family conflict), and four job resources (role clarity, job control, sense of community, predictability). Structural equation modelling (SEM) tested the JD-R framework.

**Results:**

Burnout prevalence was higher among junior (29.5%) than senior (25.1%) researchers. Job demands were associated with burnout, while job resources were associated with work engagement and buffered against burnout. Multigroup SEM did not support measurement invariance across career stages, and separate SEMs showed only moderate-to-poor fit.

**Conclusion:**

These findings suggest that the measurement of job demands and resources, as operationalized here, may not operate equivalently across career stages in this heterogeneous, multi-country sample. Career-stage-specific, longitudinal, and multilevel approaches are needed to establish measurement equivalence before testing the JD-R framework’s generalizability in academia.

## Introduction

Workplace conditions are key determinants of mental health across the global workforce [1]. While moderate stress may be adaptive, prolonged or unmanaged stress can lead to burnout, defined in ICD-11 as an occupational phenomenon resulting from chronic workplace stress and associated with increased risk of depression. Burnout is officially recognized as a compensable occupational disease in several European countries, including Denmark, France, Latvia, Portugal, and Sweden [2–5].

Although academia has traditionally been viewed as a relatively low-stress profession, growing evidence indicates high levels of occupational stress among researchers [6, 7]. A Belgian study on PhD students reported that 32% were at high risk of a common psychiatric disorder, more than twice the rate among comparably highly educated general population (14%) [8]. Early-career researchers are particularly vulnerable due to job insecurity, short-term contracts, limited funding opportunities, and delayed career stability [7, 9–14]. Across career stages, long working hours, high competition driven by performance metrics, and multiple role demands, including research, teaching, and administrative responsibilities, further contribute to a demanding work environment [15–18]. These structural challenges have prompted calls at the European level for reforms addressing precarious employment and researcher wellbeing [19].

Workplace resources play a critical role in mitigating burnout and promoting wellbeing. Access to autonomy, clear roles, supportive supervision, and a strong sense of community can enhance resilience, job satisfaction, and overall functioning [20–22]. Such resources are central to occupational health interventions aimed at preventing burnout and sustaining workforce capacity [23].

The Job Demands-Resources (JD-R) framework offers a theoretical basis for integrating these observations. The model proposes two distinct but parallel processes [24, 25]: a health-impairment process, in which excessive job demands deplete employees’ physical and psychological resources and increase the risk of burnout, and a motivational process, in which job resources foster engagement, motivation, and positive work outcomes, in part by buffering the impact of demands. This dual-process structure is why burnout and work engagement are considered jointly here, rather than as alternative outcomes: they represent the two theoretically distinct pathways the JD-R model predicts, and examining both allows a fuller test of the framework than either outcome alone would provide. The model’s core propositions have received substantial meta-analytic support across occupational contexts [26] and have been shown to generalize in direction, if not always in magnitude, across diverse employment sectors [27, 28].

Applied specifically to academia, JD-R research has linked job demands and resources to burnout and engagement among university academics [9, 29–31]. Of particular relevance to the present study, prior work suggests that the relevance of specific demands and resources may shift across career stages which found that different demands were more salient at different points in working life, while resources remained broadly protective throughout [32]. This raises the possibility that a single JD-R measurement model, calibrated without regard to career stage, may not adequately capture the experience of researchers at very different points in their careers from PhD candidates to full professors, who differ markedly in job security, autonomy, and role demands. Applying the Job Demands-Resources (JD-R) framework [24, 25], this study examines whether academic demands (job insecurity, interpersonal conflict, work-family conflict, illegitimate tasks) and resources (job control, predictability at work, role clarity, sense of community) are associated with burnout and work engagement among academic researchers, and whether this association holds equivalently across career stages (PhD candidates to full professors). Consistent with the JD-R framework’s dual-process model, we hypothesized that job demands would be positively associated with burnout, while job resources would be negatively associated with burnout and positively associated with work engagement, in both junior and senior researchers.

## Methods

### Study design and ethical considerations

This study was conducted as part of the Researcher Mental Health Observatory (ReMO), an European Union COST Action (CA19117) which aims to foster mental health and wellbeing within the European research community. The study employed a cross-sectional design.

This study was evaluated by the Ethics Commission of the Leibniz University Hannover and approved under ethics vote EV LUH 04/2023. Participants provided explicit informed consent electronically and confirmed they were ≥18 years old before proceeding. The study adheres to the principles of the Declaration of Helsinki. Participation was voluntary and anonymous [33].

### Participants and sampling

Participants were researchers working in universities and non-university research organisations across Europe. Eligibility required being ≥18 years old and holding an active research position: R1 (PhD candidates), R2 (postdoctoral researchers), R3 (senior researchers), or R4 (full professors). Students and administrative or technical staff were excluded. Recruitment used convenience and snowball sampling via mailing lists (61.2%), social media, and professional groups. Country- and institution-level breakdowns of participants were not available for reporting because the ReMO STAIRCASE data-governance agreement restricts disclosure of participant country and institutional identifiers, in order to protect the anonymity of participating institutions. A conventional response rate could not be computed because participants were recruited through multiple parallel channels (institutional and professional mailing lists, social media, worker representation groups, and word of mouth) for which no common invitation denominator exists.

### Data collection

Data were collected via the *Sustainable Working Conditions in Academia (STAIRCASE)* survey using LimeSurvey between September 2023 and August 2024. The questionnaire included five sections: demographics, mental health and wellbeing, working conditions, work environment, and leadership/supervision. For more detailed information, please see the dedicated reports [33, 34].

### Constructs considered and instruments used

The study was guided by the JD-R framework [35]. Burnout was measured using the 12-item Burnout Assessment Tool (BAT-12), a well-validated instrument capturing exhaustion, mental distance, and emotional and cognitive impairment [36–38], rated on a five-point frequency scale (1 = “never”, 5 = “always”).

Work engagement was assessed using the UWES-3 [39], a validated measure of vigor, dedication, and absorption, rated on a six-point Likert scale (1 = “never”, 6 = “always”) [40].

Job demands initially included overwork, illegitimate tasks, work-family conflict, interpersonal conflict, and job insecurity. However, overwork was excluded from the final measurement model due to an excessive proportion of missing data ( 58% ); consequently, the final job demands construct comprised illegitimate tasks, work-family conflict, interpersonal conflict, and job insecurity. Illegitimate tasks were measured using the four-item Bern Illegitimate Tasks Scale (BITS), a validated measure of unreasonable tasks [41]. Work-family conflict was assessed using a five-item validated scale capturing interference of work with family responsibilities [42]. Interpersonal conflict was measured using three items from the Knowledge-Intensive Work Environment Survey Target (KIWEST) [43]. Job insecurity was assessed using the four-item Job Insecurity Scale (JIS), validated across European contexts [44]. All job demand items were rated on five-point Likert scales (1 = “fully disagree”, 5 = “fully agree”).

Job resources initially included job control, role clarity, predictability, emotional support, and sense of community. Emotional support was subsequently excluded from the measurement model due to high missingness ( 42% ); all other retained variables exhibited missing data rates below 30% .

Job control was measured with well-validated tool of autonomy and decision-making using three items from the Job Diagnostic Survey (JDS) [45, 46], while the remaining resources were assessed using validated scales from the Copenhagen Psychosocial Questionnaire III [47–49]. All job resource items used five-point Likert scales (1 = “fully disagree”, 5 = “fully agree”).

To characterize the missing-data mechanism for these two indicators, logistic regressions were conducted predicting each item’s missingness from career stage, age category, and gender (Supplementary Table S2). Full-information maximum likelihood (FIML; the mlmv estimator in Stata) was also tested as a sensitivity analysis, retaining both indicators in the measurement model.

Apart from job insecurity, none of the items of the different measures described below were counter indicative. Higher scores on items thus generally reflected higher levels on the construct in question.

### Quantitative variables

Age was reported in categories (e.g., 25–27, 28–30) and converted to the midpoint of each interval (e.g., 26 for “25–27”) for use as a continuous covariate. Gender was coded dichotomously (0 = male, 1 = female). Civil status was recorded as a five-category variable: single; in a relationship, co-habitating; in a relationship, not co-habitating; married, co-habitating; and married, not co-habitating. Contract type was recorded as a five-category variable (contract ending within 12 months, 1–2 years, 2–3 years, or >3 years, or permanent contract).

### Estimation of burnout point-prevalence

Burnout prevalence was estimated using established BAT cut-offs (Schaufeli et al., 2023): <2.54 no burnout, ≥2.54 at risk, ≥2.96 burnout [50].

### Statistical analysis

Structural equation modelling (SEM) was used to examine relationships between job demands, job resources, burnout, and work engagement. Participants were grouped into junior (R1–R2) and senior (R3–R4) researchers.

Burnout and work engagement were modelled as latent constructs. Job demands and resources were also latent constructs represented by factor-score indicators (see Supplementary Table S1 for measurement properties and factor structures). Covariates included age and gender.

To evaluate group equivalence, a multigroup SEM approach tested measurement invariance across academic career stages (configural, metric, and scalar models). Because strict measurement invariance was not supported by the fit indices, direct cross-group parameter comparisons were deemed psychometrically invalid. Consequently, separate single-group SEM models were estimated for junior and senior researchers to allow for a cautious, exploratory examination of structural patterns within each career stage independently. Model fit was evaluated using standard global and incremental fit indices (CFI, TLI, RMSEA, SRMR). Correlated error residuals were added only where theoretically justifiable based on modification indices. Sensitivity analyses examined gender effects.

## Results

### Sample characteristics and burnout prevalence

Junior and senior researchers differed in demographic and employment characteristics (Table 1). Juniors were predominantly younger (63.1% under 34 years), whereas seniors were mainly aged 40 years and older (83.2%). Women represented the majority in both groups (63.9% juniors; 59.6% seniors). Civil status also differed, with juniors more often single (31.3%) compared to seniors (14.0%), while seniors were predominantly married (59.6% vs. 23.0% among juniors). Burnout prevalence was higher among juniors (29.5%) than seniors (25.1%), with significant group differences (χ^2^(8) = 38.40, p < 0.001).

**TABLE 1:** Demographic, professional characteristics, and burnout prevalence among junior and senior researchers (ReMO STAIRCASE survey, 27 European countries, 2023–2024).

Characteristics	Junior researchers (*n* = 2,705)	Senior researchers (*n* = 1,321)
Age		
<28	612 (22.6%)	6 (0.5%)
28–33	1,096 (40.5%)	29 (2.2%)
34–39	555 (20.5%)	182 (13.8%)
40–45	259 (9.6%)	341 (25.8%)
46–51	107 (4.0%)	297 (22.5%)
52–57	42 (1.6%)	222 (16.8%)
>57	23 (0.9%)	239 (18.1%)
Missing	11 (0.4%)	5 (0.4%)
Gender		
Female	1,729 (63.9%)	787 (59.6%)
Male	926 (34.2%)	514 (39.0%)
Missing	50 (1.8%)	20 (1.5%)
Civil status		
In a relationship, co-habitating	754 (28.5%)	194 (15.2%)
In a relationship, not co-habitating	409 (15.1%)	81 (6.1%)
Married, co-habitating	623 (23.0%)	787 (59.6%)
Married, not co-habitating	31 (1.1%)	26 (2.0%)
Single	825 (31.3%)	185 (14.0%)
Missing	63 (2.3%)	48 (3.6%)
Contract termination		
Within 12 months	855 (31.6%)	74 (5.6%)
1–2 years	517 (19.1%)	42 (3.2%)
2–3 years	420 (15.5%)	59 (4.5%)
>3 years	146 (5.4%)	60 (4.5%)
Permanent	305 (11.3%)	809 (61.2%)
Missing	462 (17.1%)	277 (21.0%)
Burnout		
No burnout	1,330 (49.2%)	783 (59.3%)
At risk	578 (21.4%)	206 (15.6%)
Burnout	797 (29.5%)	332 (25.1%)

Missingness on overwork was significantly associated with career stage (standardized β = 0.39, *p* < 0.001), whereas missingness on emotional support was not significantly associated with the observed covariates (likelihood-ratio χ^2^(8) = 10.42, *p* = 0.237). The FIML specification did not converge to a stable, identified solution (Supplementary Note under Supplementary Table S2). We therefore retained standard ML with listwise deletion as the primary estimator, with overwork and emotional support excluded from the final measurement model.

### Multigroup structural equation modelling and measurement invariance

Measurement invariance between junior and senior researchers was examined using a multigroup SEM framework. As detailed in Supplementary Table S3, the configural and metric models showed similar SRMR values (0.071 and 0.075, respectively), although several fit statistics were not reported by the software. In contrast, the scalar invariance model showed poor fit, with χ^2^(244) = 3,584.47, *p* < 0.001, RMSEA = 0.101 (90% CI: 0.098–0.104), CFI = 0.770, TLI = 0.746, and SRMR = 0.079. Thus, full scalar measurement invariance was not supported across junior and senior researchers, suggesting that item intercepts were not equivalent across academic career stages. Consequently, direct comparisons of latent factor means across groups were not considered appropriate. To avoid imposing unsupported cross-group measurement constraints, separate single-group SEMs were subsequently estimated for junior and senior researchers.

### Separate structural equation models for junior and senior researchers

Because measurement invariance was not established (Section *Multigroup structural equation modelling and measurement invariance*), separate single-group structural equation models (SEMs) were estimated for junior and senior researchers (Figures 1, 2; Tables 2, 3). Model fit was moderate to poor in both groups (Tables 2, 3). For junior researchers, SRMR = 0.07, CFI = 0.82, TLI = 0.77, and RMSEA = 0.09; corresponding values for senior researchers were 0.07, 0.84, 0.81, and 0.09, respectively. The coefficients of determination were 0.92 for junior researchers and 0.91 for senior researchers.

**FIGURE 1:**
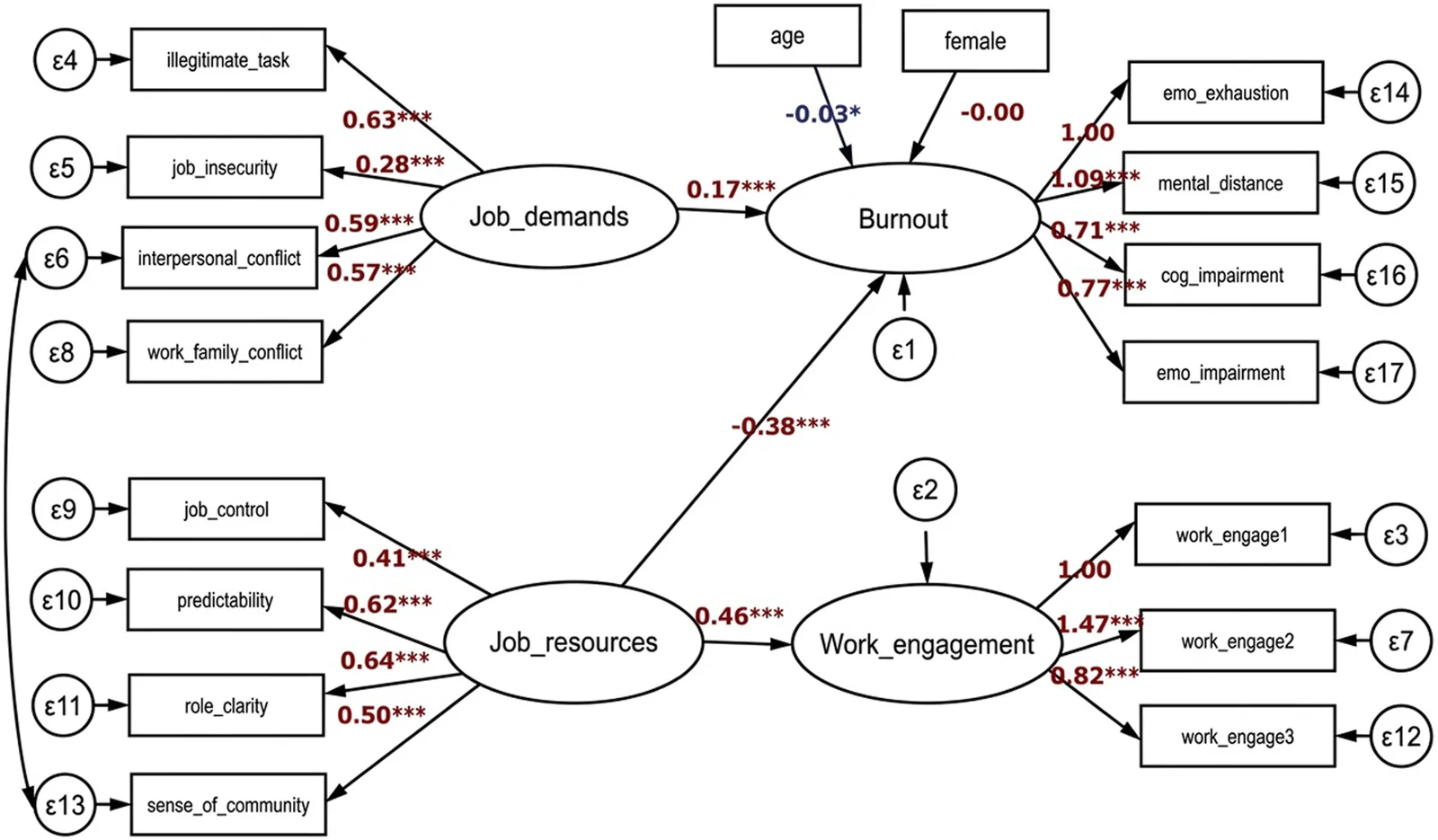
Path diagram of the job demands and resources framework among junior researchers (ReMO STAIRCASE survey, 27 European countries, 2023–2024). Note. Values in red represent structural path coefficients and factor loadings and asterisks (*) indicate significant paths. ***p < 0.001. Latent variables are represented by ovals and observed variables by rectangles. Single-headed arrows indicate hypothesized regression paths with standardized coefficients, while double-headed arrows indicate covariances.

**FIGURE 2:**
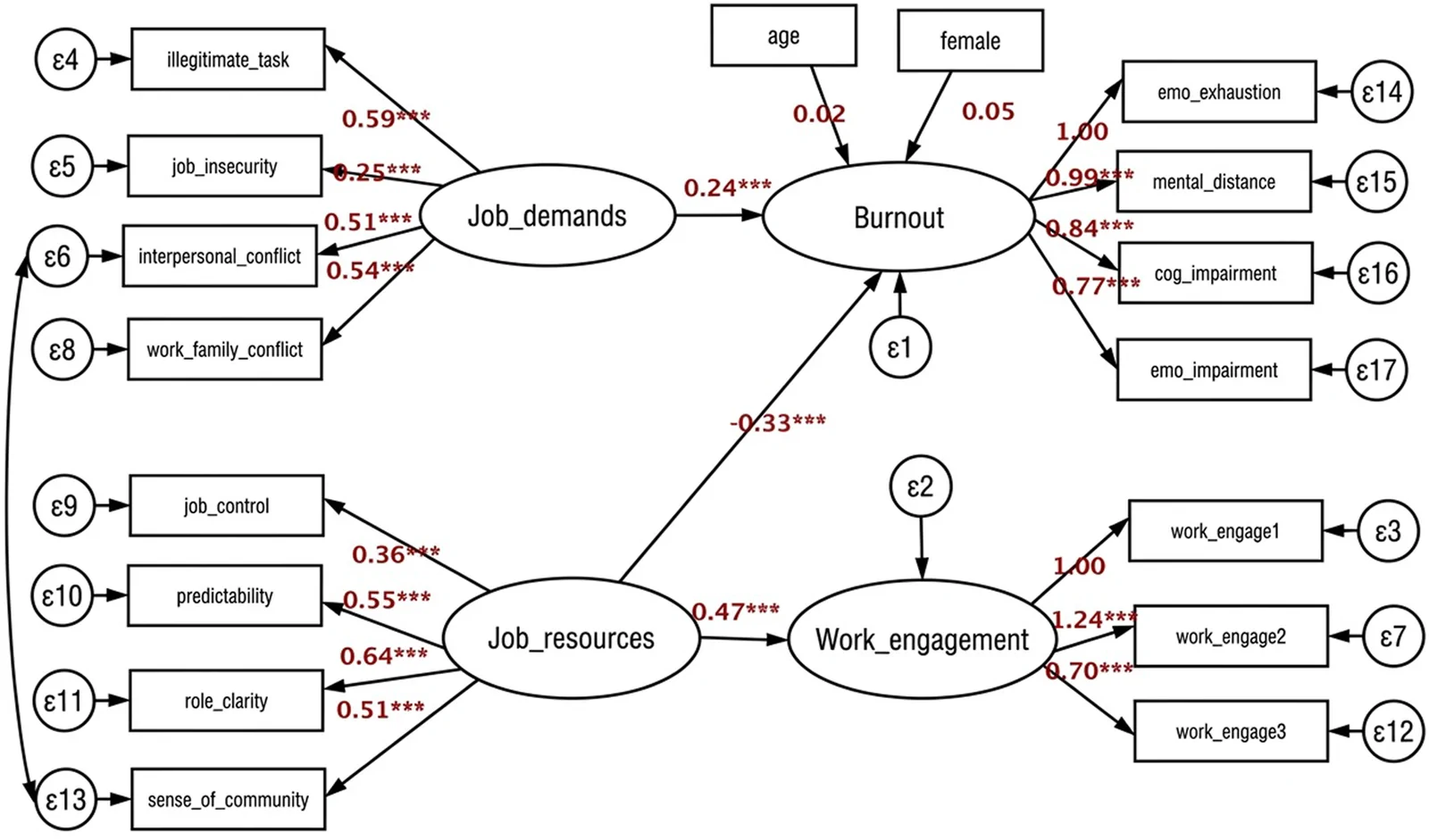
Path diagram of the job demands and resources framework among senior researchers (ReMO STAIRCASE survey, 27 European countries, 2023–2024).

**TABLE 2:** Structural model results and model fit indices for the job demands and resources framework among junior researchers, N = 1,824 (ReMO STAIRCASE survey, 27 European countries, 2023–2024).

Construct	Standardized coefficient (β)	95% CI	p-value
Panel A: Structural model results			
Age → burnout	−0.03	[−0.05, −0.01]	<0.05*
Female → burnout	−0.00	[−0.05, 0.05]	0.96
Job demand → burnout	0.17	[0.07, 0.26]	<0.001***
Job resource → burnout	−0.38	[−0.47, −0.29]	<0.001***
Job resource → work engagement	0.46	[0.41, 0.50]	<0.001***

Fit index	Value
Panel B	
χ^2^ (df = 109)	1898.45
p-value	<0.001***
RMSEA	0.09
RMSEA (90% CI)	0.09–0.10
CFI	0.82
TLI	0.77
SRMR	0.07
Coefficient of determination (CD)	0.92

CI, confidence interval; χ^2^, chi-square statistic; RMSEA, Root Mean Square Error of Approximation; CFI, Comparative Fit Index; TLI, Tucker–Lewis Index; SRMR, Standardized Root Mean Square Residual; CD, Coefficient of Determination. *p < 0.05; **p < 0.001.

**TABLE 3:** Structural model results of the job demands and resources framework among senior researchers, N = 880 (ReMO STAIRCASE survey, 27 European countries, 2023–2024).

Construct	Standardized coefficient (β)	95% CI	p-value
Panel A			
Age → burnout	0.02	[-0.01, 0.04]	0.223
Female → burnout	0.05	[-0.02, 0.13]	0.185
Job demand → burnout	0.24	[0.12, 0.36]	<0.001***
Job resource → burnout	−0.33	[-0.44, −0.22]	<0.001***
Job resource → work engagement	0.47	[0.39, 0.54]	<0.001***

Fit statistic	Value
Panel B	
Chi-square (χ^2^)	837.85
Degrees of freedom (df)	109
RMSEA	0.09
90% CI for RMSEA	[0.08, 0.09]
CFI	0.84
TLI	0.81
SRMR	0.07
Coefficient of determination (CD)	0.91

CI, confidence interval; χ^2^ = chi-square statistic; RMSEA, Root Mean Square Error of Approximation; CFI, Comparative Fit Index; TLI, Tucker–Lewis Index; SRMR, Standardized Root Mean Square Residual; CD, Coefficient of Determination. *p < 0.05; **p < 0.001.

Among junior researchers, job demands were positively associated with burnout (β = 0.17, p < 0.001), whereas job resources were negatively associated with burnout (β = −0.38, p < 0.001) and positively associated with work engagement (β = 0.46, p < 0.001). Age was negatively associated with burnout (β = −0.03, p < 0.05), whereas female gender was not associated with burnout (β = −0.00, p = 0.96). Standardized factor loadings ranged from 0.38 to 0.63 for job demands and from 0.41 to 0.64 for job resources; all freely estimated loadings for burnout and work engagement were significant (all p < 0.001; Supplementary Table S4).

Among senior researchers, job demands were positively associated with burnout (β = 0.24, p < 0.001), whereas job resources were negatively associated with burnout (β = −0.33, p < 0.001) and positively associated with work engagement (β = 0.47, p < 0.001). Neither age (β = 0.02, p = 0.223) nor female gender (β = 0.05, p = 0.185) was associated with burnout. Standardized factor loadings ranged from 0.25 to 0.59 for job demands and from 0.36 to 0.64 for job resources; all freely estimated loadings for burnout and work engagement were significant (all p < 0.001; Supplementary Table S5).

## Discussion

This study examined the applicability of a JD-R framework to burnout and work engagement among junior and senior researchers in a heterogeneous sample spanning 27 countries. The findings provide two main observations. First, measurement invariance was not established across career stages, and the separate subgroup SEMs showed moderate-to-poor fit. Second, despite these measurement and model-fit limitations, the estimated structural associations within both groups were broadly consistent with the central propositions of JD-R theory: job demands were positively associated with burnout, whereas job resources were negatively associated with burnout and positively associated with work engagement. These findings should therefore be interpreted cautiously as exploratory evidence regarding the current operationalization of the JD-R framework, rather than as evidence of differences in how the underlying theoretical relationships operate across career stages.

### Measurement invariance and multigroup SEM

The multigroup SEM did not support measurement invariance across junior and senior researchers. The configural and metric invariance models did not converge to a stable, identified solution, and only the more constrained scalar model (equal loadings and intercepts) could be estimated, showing poor fit. Thus, the measurement model did not demonstrate equivalence across career stages, limiting the basis for interpreting between-group differences in latent means [51]. Importantly, this finding does not demonstrate that the JD-R framework itself lacks generalizability to academic researchers. Rather, it indicates that the specific operationalization and SEM specification used in the present study did not function equivalently across the two groups. As a complementary strategy that does not depend on measurement invariance, a dominance analysis of the same predictor variables was conducted separately for junior and senior researchers to rank their relative contribution to explained burnout variance [52].

This distinction is important because JD-R theory is deliberately broad and flexible, allowing job demands and resources to be defined according to occupational and organizational context [24, 25, 53]. The flexibility of the framework has contributed to its applicability across diverse occupations and settings. Meta-analytic evidence supports the core JD-R relationships, showing that job demands are positively related to burnout, while job resources are negatively related to burnout and positively related to work engagement [26]. Similarly, a multi-sector study found that although employees across healthcare, industry, service, and public-sector settings differed in their levels of demands, resources, burnout, and engagement, the strength of the relationships between these constructs did not differ significantly across sectors [27]. Thus, variation in the levels or experience of job demands and resources does not necessarily imply that the underlying JD-R relationships differ across groups.

The lack of full measurement invariance may partly reflect the substantial heterogeneity of the present sample. Researchers were recruited from 27 countries and diverse academic and research contexts, while country-level clustering, institutional characteristics, and disciplinary context were not modelled. Such heterogeneity may influence how specific demands and resources are experienced and interpreted. The present analysis cannot determine whether the lack of invariance was attributable primarily to career stage, national context, institutional setting, discipline, or their combination. Accordingly, the findings are better interpreted as indicating limitations in the cross-group equivalence of the study-specific measurement model than as evidence against the generalizability of the underlying JD-R framework.

### Structural associations with burnout and work engagement

Within both career-stage groups, the estimated structural associations were broadly consistent with previous JD-R research. Among junior researchers, job demands were positively associated with burnout (β = 0.17), whereas job resources were negatively associated with burnout (β = −0.38) and positively associated with work engagement (β = 0.46). The corresponding associations among senior researchers were also in the expected direction: job demands were positively associated with burnout (β = 0.24), while job resources were negatively associated with burnout (β = −0.33) and positively associated with work engagement (β = 0.47).

The positive association between job demands and burnout is consistent with the health-impairment process proposed by JD-R theory and with previous empirical evidence. A meta-analytic test of the JD-R model found a positive association between job demands and burnout, while job resources were negatively associated with burnout; resources were also consistently positively associated with work engagement [26]. Similar findings have been reported specifically among academic staff. In a study of 595 academics in South African higher education institutions, job demands and a lack of job resources were associated with burnout, while job resources were positively associated with work engagement [29]. More recent academic research likewise indicates that psychosocial working conditions are associated with burnout among faculty, with effort increasing burnout and reward reducing it [31]. These findings are therefore broadly consistent with the directional pattern observed in the present study.

The negative association between job resources and burnout and the positive association between resources and work engagement are also consistent with the motivational processes described in JD-R research [35]. Earlier work in higher education has similarly shown that job resources such as autonomy, feedback, and other supportive conditions can play a role in protecting against burnout and supporting positive work-related outcomes. Bakker et al. found among 1,012 employees of a higher-education institution that job resources could buffer the effects of job demands on burnout, although the buffering effect varied across specific demand-resource combinations [54].

The estimated structural associations were broadly similar in direction across the two career-stage groups, consistent with the central propositions of JD-R theory. However, given the lack of full measurement invariance and the poor fit of the subgroup models, the estimates should be interpreted cautiously within each group and should not be used to infer differences in the strength of these associations between junior and senior researchers. Previous research suggests that the relevance of specific job demands and resources may vary across career stages which found that different demands were associated with burnout at different career stages, while job resources were associated with engagement across stages [32]. Although such findings make career-stage variation theoretically plausible, the present study does not provide a formal test of such differences.

### Specific job demands in the academic context

The measurement results indicate that several demands contributed to the job demands construct in both groups, including work-family conflict, interpersonal conflict, illegitimate tasks, and job insecurity. These findings are consistent with previous research showing that academic work involves a combination of quantitative, organizational, and work-family demands [30].

Work-family conflict has received particular attention in relation to academic burnout. A study of 2,229 university faculty members in the Czech Republic found that work-family conflict was strongly associated with burnout within a JD-R-based model that also included quantitative demands, job insecurity, and several job resources [55]. Related research likewise identifies work–family conflict as an important correlate of burnout in academic settings [55–57]. The present findings are consistent with this literature, although the current model treated work-family conflict as an indicator of a broader latent job-demands construct and therefore did not test it as an individual predictor of burnout.

Illegitimate tasks also contributed substantially to job demands in both groups. These refer to duties perceived as unnecessary or unreasonable because they fall outside what an employee believes can legitimately be expected within their professional role [58, 59], and have been linked to burnout and strain, including variance in burnout beyond other workplace stressors [60]. Research among doctoral and postdoctoral researchers has similarly linked illegitimate tasks to burnout [61]. While senior researchers sometimes engage in such tasks, they are also more focused on higher-level responsibilities such as leadership, grant management, and dissemination [62–64].

Interpersonal conflict and job insecurity likewise contributed to the broader job-demands construct and may be particularly salient in academic settings marked by hierarchical relationships, resource competition, and employment uncertainty; 38.1% of junior researchers in the present sample held contracts shorter than 1 year. Prior research links precarious academic employment to reduced autonomy and psychological security [65–67], consistent with broader uncertainty in European research funding [68–70]. Because job insecurity and interpersonal conflict were modelled as indicators rather than separate predictors, however, the present analysis cannot establish their independent associations with burnout.

### Specific job resources in the academic context

Role clarity showed a relatively high loading on the job resources construct in both career-stage groups. This is consistent with previous academic research in which role clarity was included as a job resource and was associated with lower burnout among university faculty [55]. The convergence suggests that clarity regarding responsibilities and expectations may be an important component of the psychosocial resource environment in academia. Nevertheless, the loading indicates the contribution of role clarity to the latent job-resources construct; it should not be interpreted as evidence that role clarity itself was a stronger predictor of burnout than the other resources.

Sense of community was also included as an indicator of job resources in the present model. This is consistent with research on academic researchers showing that a sense of belonging to a scholarly or research community is a well-established requirement for researchers’ development and wellbeing, with isolation and lack of community linked to greater attrition and disengagement [71]. These findings reinforce the theoretical relevance of a supportive research community as a resource, but they do not allow reliable conclusions about whether sense of community matters more for junior than senior researchers.

### Model fit of the separate subgroup SEMs

The separate SEMs showed moderate-to-poor fit in both career-stage groups, indicating that the specified models did not fully reproduce the observed covariance structure. This is consistent with the sample heterogeneity and framework flexibility discussed in Section *Measurement invariance and multigroup SEM*, which likely contributed to both the lack of measurement invariance and the imperfect fit of the subgroup models.

Model fit may additionally have been influenced by the measurement specification itself. Job demands and job resources were represented using factor-score indicators, whereas burnout and work engagement were modelled using item-level latent indicators. In addition, overwork and emotional support were excluded because of missingness, potentially narrowing the content coverage of the respective constructs.

Importantly, the poor fit of the subgroup models should not be interpreted as evidence that JD-R theory is inadequate for understanding academic wellbeing. A comprehensive review of JD-R research supports the relevance of the framework to burnout and work engagement across diverse occupational settings [28]. Given the poor model fit, the estimated structural associations should be interpreted cautiously, even within each subgroup, and not as evidence of systematic differences between junior and senior researchers.

### Limitations and strengths of the study

Several limitations should be considered. First, the cross-sectional design prevents causal inference and cannot rule out reverse causation (e.g., burnout may itself increase perceived demands or reduce access to resources), and self-reported measures may introduce common-method bias. Second, convenience and snowball sampling across 27 countries limits representativeness; country- and institution-level composition could not be reported due to data-governance restrictions, and country, institutional, and disciplinary clustering were not modelled, which may have contributed to the measurement and fit problems observed. Third, as noted above, measurement invariance was not established and the subgroup SEMs showed moderate-to-poor fit, so the structural coefficients remain exploratory. Finally, missingness constrained the operationalization of job demands and resources, including the exclusion of overwork and emotional support; a sensitivity analysis using full-information maximum likelihood did not converge, supporting this exclusion-based approach.

The study also has important strengths. The large sample, cross-national coverage, use of established measures, and explicit evaluation of measurement invariance provide a valuable assessment of the challenges involved in applying the JD-R framework to academic researchers. A further strength was that measurement equivalence across career stages was formally tested rather than assumed. Because full invariance was not established, separate SEMs were estimated for junior and senior researchers without imposing unsupported equality constraints across groups.

### Implications for future research

Future research should use longitudinal and multilevel designs to examine variation in JD-R processes across career stages while accounting for country, institutional, disciplinary, and employment contexts. Measurement invariance should be established before making substantive cross-group comparisons, with formal tests of between-group parameter differences conducted where the relevant invariance assumptions are supported. More context-specific operationalizations of job demands and resources may also be warranted, given evidence that the relevance of particular demands and resources can vary across working life [32]. Such approaches would help distinguish potential career-stage effects from broader contextual heterogeneity. Future research should also examine whether JD-R processes vary by academic discipline, given differing workload structures and funding pressures across fields, and whether socio-demographic factors such as membership in an equity-seeking group moderate relationships between job demands, resources, and burnout; neither could be examined in the present study.

### Conclusion

The present study did not establish full scalar measurement invariance across junior and senior researchers, and the separate subgroup models showed moderate-to-poor fit; the structural estimates should therefore be interpreted cautiously and not as evidence of true differences between career stages. Nevertheless, the directions of the estimated associations were consistent with JD-R theory in both groups: job demands were positively associated with burnout, while job resources were negatively associated with burnout and positively associated with work engagement. These findings reflect challenges in applying a common JD-R measurement model to a highly heterogeneous, multi-country sample, rather than evidence that the JD-R framework itself operates differently across career stages, and point to the need for longitudinal, multilevel, context-sensitive research before career-stage differences can be established.

## References

[1] World Health Organization (WHO). Mental Health at Work. Geneva: World Health Organization (2024). Available online at: https://www.who.int/news-room/fact-sheets/detail/mental-health-at-work (Accessed October 5, 2026).

[2] Maslach C Jackson SE . The measurement of experienced burnout. J Occup Behav (1981) 2:99–113. 10.1002/job.4030020205

[3] Maslach C Schaufeli WB Leiter MP . Job burnout. Annu Rev Psychol (2001) 52:397–422. 10.1146/annurev.psych.52.1.397 11148311

[4] Guseva Canu I Marca SC Dell'Oro F Balázs Á Bergamaschi E Besse C Harmonized definition of occupational burnout: a systematic review, semantic analysis, and Delphi consensus in 29 countries. Scand J Work Environ Health (2021) 47(2):95–107. 10.5271/sjweh.3935 PMC8114565 33258478

[5] Lastovkova A Carder M Rasmussen HM Sjoberg L Groene GJ Sauni R Burnout syndrome as an occupational disease in the European Union: an exploratory study. Ind Health (2018) 56(2):160–5. 10.2486/indhealth.2017-0132 29109358 PMC5889935

[6] Bozeman B Youtie J . Robotic bureaucracy: administrative burden and red tape in university research. Public Adm Rev (2020) 80(1):157–62. 10.1111/puar.13105

[7] Reevy GM Deason G . Predictors of depression, stress, and anxiety among non-tenure track faculty. Front Psychology (2014) 5:701. 10.3389/fpsyg.2014.00701 25071667 PMC4085875

[8] Levecque K Anseel F De Beuckelaer A Van der Heyden J Gisle L . Work organization and mental health problems in PhD students. Res Pol (2017) 46(4):868–79. 10.1016/j.respol.2017.02.008

[9] Boyd CM Bakker AB Pignata S Winefield AH Gillespie N Stough C . A longitudinal test of the job demands‐resources model among Australian university academics. Appl Psychology (2011) 60(1):112–40. 10.1111/j.1464-0597.2010.00429.x

[10] Petersen AM Riccaboni M Stanley HE Pammolli F . Persistence and uncertainty in the academic career. Proc Natl Acad Sci (2012) 109(14):5213–8. 10.1073/pnas.1121429109 22431620 PMC3325726

[11] Acker S Haque E . The struggle to make sense of doctoral study. Higher Educ Res and Develop (2015) 34(2):229–41. 10.1080/07294360.2014.956699

[12] Barry KM Woods M Warnecke E Stirling C Martin A . Psychological health of doctoral candidates, study-related challenges and perceived performance. Higher Educ Res and Develop (2018) 37(3):468–83. 10.1080/07294360.2018.1425979

[13] Nagy GA Fang CM Hish AJ Kelly L Nicchitta CV Dzirasa K Burnout and mental health problems in biomedical doctoral students. CBE—Life Sci Educ (2019) 18(2):ar27. 10.1187/cbe.18-09-0198 31150319 PMC6755223

[14] Initiative for Science in Europe (ISE). Position on Precarity of Academic Careers. Brussels: Initiative for Science in Europe (2021). Available online at: https://initiative-se.eu/2021/02/05/precarity-paper-2021/ (Accessed October 5, 2026).

[15] Woolston C . PhDs: the tortuous truth. Nature (2019) 575(7782):403–6. 10.1038/d41586-019-03459-7 31723297

[16] Miller AN Taylor SG Bedeian AG . Publish or perish: academic life as management faculty live it. Career Development International (2011) 16(5):422–45. 10.1108/13620431111167751

[17] Waaijer CJ Sonneveld H Buitendijk SE van Bochove CA van der Weijden IC . The role of gender in the employment, career perception and research performance of recent PhD graduates from Dutch universities. PLoS One (2016) 11(10):e0164784. 10.1371/journal.pone.0164784 27736968 PMC5063396

[18] Cho S Hayter CS . Under pressure: a systematic review of stress and its impact among graduate students. Sci Public Pol (2020) 47(6):758–71. 10.1093/scipol/scaa053

[19] Council of EU. Improving conditions for research careers in Europe: Council adopts conclusions [press release]. European council. Improving conditions for research careers in Europe: Council adopts conclusions - Consilium. Brussels: Council of the European Union (2021).

[20] Laschinger HKS Finegan JE Shamian J Wilk P . A longitudinal analysis of the impact of workplace empowerment on work satisfaction. J Organizational Behav The Int J Ind Occup Organizational Psychol Behav (2004) 25(4):527–45. 10.1002/job.256

[21] Laschinger HK Wong CA Greco P . The impact of staff nurse empowerment on person-job fit and work engagement/burnout. Nurs Adm Q (2006) 30(4):358–67. 10.1097/00006216-200610000-00008 17077717

[22] Nedd N . Perceptions of empowerment and intent to stay. Nurs Econ (2006) 24(1):13–8. 16583601

[23] Kanter RM . Power failure in management circuits. In: Leadership Perspectives. London: Routledge (2017). p. 281–90.

[24] Demerouti E Bakker AB Nachreiner F Schaufeli WB . The job demands-resources model of burnout. J Appl Psychol (2001) 86(3):499–512. 10.1037/0021-9010.86.3.499 11419809

[25] Bakker AB Demerouti E . The job demands-resources model: state of the art. J Managerial Psychol (2007) 22(3):309–28. 10.1108/02683940710733115

[26] Crawford ER LePine JA Rich BL . Linking job demands and resources to employee engagement and burnout: a theoretical extension and meta-analytic test. J Appl Psychol (2010) 95(5):834–48. 10.1037/a0019364 20836586

[27] Van den Broeck A Vander Elst T Baillien E Sercu M Schouteden M De Witte H Job demands, job resources, burnout, work engagement, and their relationships: an analysis across sectors. J Occup Environ Med (2017) 59(4):369–76. 10.1097/JOM.0000000000000964 28157768

[28] Bakker AB Demerouti E . Job demands-resources theory: taking stock and looking forward. J Occup Health Psychol (2017) 22(3):273–85. 10.1037/ocp0000056 27732008

[29] Barkhuizen N Rothmann S van de Vijver FJ . Burnout and work engagement of academics in higher education institutions: effects of dispositional optimism. Stress Health (2014) 30(4):322–32. 10.1002/smi.2520 23949954

[30] Naidoo-Chetty M du Plessis M . Job demands and job resources of academics in higher education. Front Psychol (2021) 12:631171. 10.3389/fpsyg.2021.631171 PMC8260971 34248738

[31] Tanimoto AS Richter A Lindfors P . How do effort, reward, and their combined effects predict burnout, self-rated health, and work-family conflict among permanent and fixed-term faculty? Ann Work Expo Health (2023) 67(4):462–72. 10.1093/annweh/wxac094 PMC10119699 36617194

[32] Salmela-Aro K Upadyaya K . Role of demands-resources in work engagement and burnout in different career stages. J Vocational Behav (2018) 108:190–200. 10.1016/j.jvb.2018.08.002

[33] Lasser J Mol ST Čontala A Slavec A de Swarte AZ Khachatryan A Data from the researcher mental health observatory STAIRCASE survey. J Open Psychol Data (2026) 14:1. 10.5334/jopd.136 PMC13220731 42222125

[34] Keopraseuth L Gomez Ortiz A Wild P Guseva Canu I . Working conditions and mental health among Swiss researchers. Lausanne: Unisanté (2025) Raisons de santé 371. 10.16908/issn.1660-7104/371

[35] Schaufeli WB . Applying the job demands-resources model: a ‘how to’ guide to measuring and tackling work engagement and burnout. Organizational Dyn (2017) 46(2):120–32. 10.1016/j.orgdyn.2017.04.008

[36] De Beer LT Christensen M Sørengaard TA Innstrand ST Schaufeli WB . The psychometric properties of the burnout assessment tool in Norway: a thorough investigation into construct-relevant multidimensionality. Scand J Psychol (2024) 65(3):479–89. 10.1111/sjop.12996 38146078

[37] Shoman Y Hostettler R Guseva Canu I . Psychometric validity of the Shirom-Melamed Burnout Measure and the Burnout assessment tool: a systematic review. Arh Hig Rada Toksikol (2023) 74(4):238–45. 10.2478/aiht-2023-74-3769 PMC10750325 38146759

[38] Henry J Nerhot A Remy V Guseva Canu I . Validation of burnout assessment tool in French, German, and italian-speaking bus drivers: content, structure, construct and cross-cultural validity assessment in the trapheac Study. SSRN [Preprint] (2025). 10.2139/ssrn.5365651

[39] Schaufeli WB Shimazu A Hakanen J Salanova M De Witte H . An ultra-short measure for work engagement: the UWES-3 validation across five countries. Eur. J. Psychol. Assess. (2019) 35(4):577–91. 10.1027/1015-5759/a000430

[40] Schaufeli WB Bakker AB Salanova M . The measurement of work engagement with a short Questionnaire: a cross‐national study. Educ Psychol Meas (2006) 66:701–16. 10.1177/0013164405282471

[41] Basinska BA Dåderman AM . Psychometric properties of the Bern illegitimate tasks scale using classical test and item response theories. Scientific Rep (2023) 13(1):7211. 10.1038/s41598-023-34006-0 PMC10156715 37137932

[42] Netemeyer RG Boles JS McMurrian R . Development and validation of work–family conflict and family–work conflict scales. J Appl Psychol (1996) 81(4):400–10. 10.1037/0021-9010.81.4.400

[43] Innstrand ST Christensen M Undebakke KG Svarva K . The presentation and preliminary validation of KIWEST using a large sample of Norwegian university staff. Scand J Public Health (2015) 43(8):855–66. 10.1177/1403494815600562 26275640

[44] Vander Elst T De Witte H De Cuyper N . The job insecurity scale: a psychometric evaluation across five European countries. Eur J Work Organizational Psychol (2014) 23:364–80. 10.1080/1359432X.2012.745989

[45] Taber TD Taylor E . A review and evaluation of the psychometric properties of the job diagnostic survey. Personnel Psychol (1990) 43(3):467–500. 10.1111/j.1744-6570.1990.tb02393.x

[46] Idaszak JR Drasgow F . A revision of the job diagnostic survey: elimination of a measurement artifact. J Appl Psychol (1987) 72(1):69–74. 10.1037/0021-9010.72.1.69

[47] Rahimi M Arnold B LaMontagne AD Riley P . Validation and benchmarks for the copenhagen psychosocial questionnaire (COPSOQ III) in an Australian working population sample. BMC Public Health (2025) 25(1):830. 10.1186/s12889-025-21845-x PMC11871671 40025491

[48] Lincke HJ Vomstein M Lindner A Nolle I Häberle N Haug A COPSOQ III in Germany: validation of a standard instrument to measure psychosocial factors at work. J Occup Med Toxicol (2021) 16(1):50. 10.1186/s12995-021-00331-1 PMC8594291 34784940

[49] Berthelsen H Westerlund H Pejtersen JH Hadzibajramovic E . Construct validity of a global scale for workplace social capital based on COPSOQ III. PLOS ONE (2019) 14(8):e0221893. 10.1371/journal.pone.0221893 31465500 PMC6715184

[50] Schaufeli WB De Witte H Hakanen JJ Kaltiainen J Kok R . How to assess severe burnout? Cutoff points for the Burnout Assessment Tool (BAT) based on three European samples. Scand J Work Environ Health (2023) 49(4):293–302. 10.5271/sjweh.4093 37042446 PMC10713992

[51] Chen H Liu F Pang L Liu F Fang T Wen Y Are you tired of working amid the pandemic? The role of professional identity and job satisfaction against job burnout. Int Journal Environmental Research Public Health (2020) 17(24):9188. 10.3390/ijerph17249188 33316964 PMC7764790

[52] Noe MTN Wild P Mol ST Guseva Canu I . Relative importance of psychosocial and organizational predictors of burnout among European researchers: a dominance analysis. Front Public Health (2026). In press.

[53] Schaufeli WB Taris TW . In: A Critical Review of the Job demands-resources Model: Implications for Improving Work and Health. Bridging Occupational, Organizational and Public Health: A Transdisciplinary Approach. New York, NY, US: Springer Science + Business Media (2014). p. 43–68. 10.1007/978-94-007-5640-3_4

[54] Bakker AB Demerouti E Euwema MC . Job resources buffer the impact of job demands on burnout. J Occup Health Psychol (2005) 10(2):170–80. 10.1037/1076-8998.10.2.170 15826226

[55] Zabrodska K Mudrak J Solcova I Květon P Blatný M Machovcova K . Burnout among university faculty: the central role of work–family conflict. Educ Psychol (2017) 38:800–19. 10.1080/01443410.2017.1340590

[56] Allen TD Herst DEL Bruck CS Sutton M . Consequences associated with work-to-family conflict: a review and agenda for future research. J Occup Health Psychol (2000) 5(2):278–308. 10.1037/1076-8998.5.2.278 10784291

[57] Bao X Dong J Guo J . Family-to-Work conflict and innovative work behavior among university teachers: the mediating effect of work stress and the moderating effect of gender. Behav Sci (2025) 15(10):1309. 10.3390/bs15101309 41153100 PMC12561553

[58] Semmer NK Tschan F Jacobshagen N Beehr TA Elfering A Kälin W Stress as offense to self: a promising approach comes of age. Occup Health Sci (2019) 3(3):205–38. 10.1007/s41542-019-00041-5 32647746 PMC7328775

[59] Jacobshagen N . Illegitimate Tasks, Illegitimate Stressors: Testing a New stressor-strain Concept [Dissertation]. Bern, Switzerland: University of Bern (2006).

[60] Moncayo-Rizzo J Alvarado-Villa G Cossio-Uribe C . The impact of illegitimate tasks on burnout syndrome in a healthcare system: a cross-sectional study. Int J Nurs Stud Adv (2024) 6:100185. 10.1016/j.ijnsa.2024.100185 38746820 PMC11080370

[61] Julmi C Pereira JM Bramlage JK Jackenkroll B . Explaining the relationship between ethical leadership and burnout facets in the academic context: the mediating role of illegitimate tasks. Int J Organ Theor and Behav (2022) 25(12):39–55. 10.1108/IJOTB-11-2020-0204

[62] Cadena-Povea H Hernández-Martínez M Bastidas-Amador G Torres-Andrade H . What pushes university professors to burnout? A systematic review of sociodemographic and psychosocial determinants. Int J Environ Res Public Health (2025) 22(8):1214. 10.3390/ijerph22081214 40869800 PMC12386177

[63] Baruch Y . Careers in academe: the academic labour market as an eco‐system. Career Develop Int (2013) 18(2):196–210. 10.1108/CDI-09-2012-0092

[64] Mantai L Marrone M . Academic career progression from early career researcher to professor: what can we learn from job ads. Stud Higher Education (2023) 48(6):797–812. 10.1080/03075079.2023.2167974

[65] Zhao X Ding S . Phenomenology of burnout syndrome and connection thereof with coping strategies and defense mechanisms among university professors. Eur J Invest Health Psychol Educ (2019) 10(1):82–93. 10.3390/ejihpe10010008 PMC8314242 34542471

[66] Myrzabayev A Kabdolla Y Tnimova G Sokolova G Bodeyev M Sivolobova O The pre-nosological diagnostics of integral health of a university professor. Bangladesh J Med Sci (2023) 22(2):432–41. 10.3329/bjms.v22i2.65008

[67] Huamani PLT Pecho MT Palacios EEP . Predictors of Burnout syndrome in university professors: an exploratory factor análisis. Enfermería Glob (2022) 21(3):66–80. 10.6018/eglobal.496901

[68] Swiss National Science Foundation (SNSF). Federal budget cuts: SNSF to adapt its research funding from 2026. In: Federal Budget Cuts: Impact on the SNSF (2025). [press release]. https://www.snf.ch/en/Uo5KXN2tSEFzDqge/news/federal-budget-cuts-snsf-to-adapt-its-research-funding-from-2026 (Accessed October 5, 2026).

[69] Nature. The EU’s Horizon research fund needs to stay a defence‐free zone. Nature (2025), 646:260. 10.1038/d41586-025-03221-2 41057581

[70] Tollefson J . Grant cuts, arrests, lay‐offs: Trump made 2025 a tumultuous year for science. Nature (2025) 648:531. 10.1038/d41586-025-04051-y 41398065

[71] Mantai L . ‘A Source of Sanity’: the Role of Social Support for Doctoral Candidates’ belonging and becoming. Int J Doctoral Stud (2019) 14:367–82. 10.28945/4275

