# Supplementary material for: Do job demands and resources generalize across academic career stages? Implications for burnout and work engagement
Source: Int J Public Health. 2026 Oct 12;71:1610023. doi: 10.3389/ijph.2026.1610023 (PMC13696366; doi:10.3389/ijph.2026.1610023)
Supplement: Supplementary file 1 [file Supplementaryfile1.docx]

***Supplementary table S1: Constructs and instrument used for their measurement considered in factor scores for SEM analysis***

| **Construct** | **Questionnaire in REMO**  **(STAIRCASE Survey)** | **Instrument** | **Score used in SEM** |
| --- | --- | --- | --- |
| Work engagement | 1-At my work, I feel bursting with energy. | UWES (Utrecht Work Engagement Scale) | Full latent factor |
|  | 2-I am enthusiastic about my job. |  |  |
|  | 3- I am immersed in my work. |  |  |
| Burnout | 1-At work, I feel mentally exhausted | BAT-12 (Burnout Assessment Tool) | Full latent factor |
|  | 2-After a day at work, I find it hard to recover my energy. |  |  |
|  | 3-At work, I feel physically exhausted |  |  |
|  | 4-I struggle to find any enthusiasm for my work. |  |  |
|  | 5-I feel a strong aversion towards my job. |  |  |
|  | 6-I’m cynical about what my work means to others. |  |  |
|  | 7-At work, I have trouble staying focused. |  |  |
|  | 8-When I’m working, I have trouble concentrating. |  |  |
|  | 9-I make mistakes in my work because I have my mind on other things. |  |  |
|  | 10-I do not recognize myself in the way I react emotionally at work. |  |  |
|  | 11-At work I may overreact unintentionally. |  |  |
|  | 12-At work, I feel unable to control my emotions. |  |  |
| Predictability | 1-At your main place of work/study, are you informed well in advance concerning important decisions, changes, or plans for the future? | COPSOQ-III (Copenhagen Psychosocial Questionnaire) | Weighted factor score (from CFA) |
|  | 2-Do you receive all the information you need in order to do your work/study well? |  |  |
|  | 3-Do you often receive contradictory information? |  |  |
| Role clarity | 1-Do you know exactly how much say you have at work? | COPSOQ-III (Copenhagen Psychosocial Questionnaire) | Weighted factor score (from CFA) |
|  | 2-Does your work have clear objectives? |  |  |
|  | 3-Do you know exactly which areas are your responsibility? |  |  |
|  | 4-Do you know exactly what is expected of you at work? |  |  |
| Job insecurity | 1-Chances are, I will soon lose my job. | Job Insecurity Scale | Weighted factor score (from CFA) |
|  | 2-I am sure I can keep my job. |  |  |
|  | 3-I feel insecure about the future of my job. |  |  |
|  | 4-I think I might lose my job in the near future. |  |  |
| Job control | 1-I decide on my own how to go about doing the work. | Job diagnostic survey | Weighted factor score (from CFA) |
|  | 2-The job gives me a chance to use my personal initiative or judgment in carrying out the work. |  |  |
|  | 3-The job gives me considerable opportunity for independence and freedom in how I do the work. |  |  |
| Interpersonal conflict | 1-My work is hampered by power struggles and territorial thinking in my unit. | Knowledge-intensive work environment survey target (KIWEST) | Weighted factor score (from CFA) |
|  | 2-In my unit, intrigues impair the work climate. |  |  |
|  | 3-In my unit, there is a great deal of tension due to prestige and conflicts. |  |  |
| Work-family conflict | 1-The demands of my work interfere with my home and family life. | Work-family life conflict | Weighted factor score (from CFA) |
|  | 2-The amount of time my job takes up makes it difficult to fulfil family responsibilities. |  |  |
|  | 3-Things I want to do at home do not get done because of the demands my job puts on me. |  |  |
|  | 4-My job produces strain that makes it difficult to fulfil family duties. |  |  |
|  | 5-Due to work-related duties, I have to make changes to my plans for family activities. |  |  |
| Sense of community | 1-Is there a good atmosphere between you and your colleagues? | COPSOQ-III (Copenhagen Psychosocial Questionnaire) | Weighted factor score (from CFA) |
|  | 2-Is there good cooperation between the colleagues at work? |  |  |
|  | 3-Do you feel part of a community at your place of work? |  |  |
| Illegitimate tasks | Do you have work tasks to take care of, which you believe… | Bern Illegitimate Tasks Scale (BITS) | Weighted factor score (from CFA) |
|  | 1-…should be done by someone else? |  |  |
|  | 2-…are going too far, and should not be expected from you? |  |  |
|  | 3-…put you into an awkward position? |  |  |
|  | 4-…are unfair for you to have to deal with? |  |  |
|  |  |  |  |
|  |  |  |  |
|  |  |  |  |
|  |  |  |  |
|  |  |  |  |
|  |  |  |  |

***Supplementary Table S2: Logistic regression of item missingness on career stage, age, and gender (N = 3,942)***

| **Predictor** | **Missing: Overwork β (SE)** | **p** | **Missing: Emotional support β (SE)** | **p** |
| --- | --- | --- | --- | --- |
| Career stage (junior = ref) | 0.385 (0.101) | <.001 | 0.059 (0.096) | 0.540 |
| Age 28–33 | −0.283 (0.104) | 0.006 | 0.034 (0.102) | 0.739 |
| Age 34–39 | −0.502 (0.118) | <.001 | −0.066 (0.113) | 0.562 |
| Age 40–45 | −0.617 (0.135) | <.001 | −0.048 (0.129) | 0.708 |
| Age 46–51 | −0.556 (0.154) | <.001 | −0.263 (0.149) | 0.072 |
| Age 52–57 | −0.418 (0.174) | 0.007 | −0.140 (0.169) | 0.410 |
| Age >57 | −0.080 (0.176) | 0.649 | 0.194 (0.173) | 0.260 |
| Female | 0.001 (0.070) | 0.249 | −0.012 (0.067) | 0.863 |
| Constant | −0.398 (0.093) | <.001 | −0.203 (0.091) | 0.026 |
| Model χ²(8) | 47.74 | <.001 | 10.42 | 0.237 |
| Pseudo R² | 0.0093 | — | 0.0019 | — |

*Note: β = coefficient from logistic regression predicting missingness (1 = missing, 0 = observed). Reference categories: junior career stage, age <28, male.*

**Supplementary Note. FIML sensitivity analysis**

Full-information maximum likelihood (method (mlmv) in Stata) was tested as an alternative to listwise-deletion ML, reinstating overwork and emotional support as indicators. The conditional model exhibited convergence difficulties (non-concave log-likelihood iterations) and produced a non-identified solution for the job insecurity indicator, a near-zero factor loading (β ≈ 0.000002 to 0.000004, p > .5) and a boundary residual variance, in both the junior and senior subgroup models. Because this specification did not reach a stable, interpretable solution, results are not reported, and listwise-deletion ML was retained as the primary analysis.

***Supplementary table S3. Fit indices for configural, metric, and scalar invariance models in multigroup SEM (N = 2,704)***

| **Fit Statistic** | **Configural invariance (Baseline)** | **Metric invariance**  **(Δ vs Configural)** | **Scalar invariance**  **(Δ vs Metric)** |
| --- | --- | --- | --- |
| χ² (df) | Not reported | Not reported | 3,584.47 (244) |
| p-value | — | — | <0.001 |
| RMSEA | Not reported | Not reported | 0.101  (90% CI: 0.098-0.104) |
| CFI | 1.000 | 1.000 | 0.770 |
| TLI | Not reported | Not reported | 0.746 |
| SRMR | 0.071 | 0.075 | 0.079 |
| AIC | 109,473.08 | 109,488.74 | 110,269.25 |
| BIC | 110,134.16 | 110,073.09 | 110,776.87 |
| CD | 0.921 | 0.919 | 0.921 |

*Note. Configural = baseline model; Metric = equal factor loadings; Scalar = equal loadings and intercepts. RMSEA = Root Mean Square Error of Approximation; CFI = Comparative Fit Index; TLI = Tucker–Lewis Index; SRMR = Standardized Root Mean Square Residual; AIC = Akaike Information Criterion; BIC = Bayesian Information Criterion. χ², degrees of freedom, RMSEA, and TLI were not reported by the software for the configural and metric invariance models and are therefore presented as "Not reported."*

***Supplementary table S4. Measurement model results of the job demands and resources framework among junior researchers, N = 1,824 (27 European countries, 2023-2024)***

| **Latent Construct** | **Indicator** | **Standardized Loading (λ)** | **95% CI** | **p-value** |
| --- | --- | --- | --- | --- |
| Job Demands | Job insecurity | 0.38 | [0.34, 0.43] | <0.001*** |
|  | Work-family conflict | 0.57 | [0.52, 0.61] | <0.001*** |
|  | Interpersonal conflict | 0.59 | [0.54, 0.63] | <0.001*** |
|  | Illegitimate tasks | 0.63 | [0.59, 0.67] | <0.001*** |
| Job Resources | Job control | 0.41 | [0.36, 0.45] | <0.001*** |
|  | Sense of community | 0.50 | [0.46, 0.54] | <0.001*** |
|  | Predictability | 0.62 | [0.58, 0.66] | <0.001*** |
|  | Role clarity | 0.64 | [0.60, 0.68] | <0.001*** |
| Burnout | Exhaustion | 1.00 (ref) | - | Constrained |
|  | Mental distance | 1.09 | [1.01, 1.16] | <0.001*** |
|  | Cognitive impairment | 0.71 | [0.64, 0.78] | <0.001*** |
|  | Emotional impairment | 0.77 | [0.69, 0.84] | <0.001*** |
| Work Engagement | UWES Item 1 | 1.00 (ref) | — | Constrained |
|  | UWES Item 2 | 1.47 | [1.36, 1.59] | <0.001*** |
|  | UWES Item 3 | 0.82 | [0.74, 0.89] | <0.001*** |

***Note.*** *λ = standardized factor loading. ref=reference, ***p < 0.001.*

***Supplementary table S5.. Measurement model results of the job demands and resources framework among senior researchers, N = 880 (27 European countries, 2023-2024)***

| **Latent Construct** | **Indicator** | **Standardized Loading (λ)** | **95% CI** | **p-value** |
| --- | --- | --- | --- | --- |
| Job Demands | Job Insecurity | 0.25* | [0.20, 0.31] | <0.001*** |
|  | Work–Family Conflict | 0.54 | [0.47, 0.60] | <0.001*** |
|  | Interpersonal Conflict | 0.51 | [0.44, 0.57] | <0.001*** |
|  | Illegitimate Tasks | 0.59 | [0.53, 0.65] | <0.001*** |
| Job Resources | Job Control | 0.36 | [0.30, 0.42] | <0.001*** |
|  | Sense of Community | 0.51 | [0.45, 0.57] | <0.001*** |
|  | Predictability | 0.55 | [0.50, 0.60] | <0.001*** |
|  | Role Clarity | 0.64 | [0.59, 0.70] | <0.001*** |
| Burnout | Exhaustion | 1.00 (ref) | **—** | **—** |
|  | Mental Distance | 0.99 | [0.89, 1.08] | <0.001*** |
|  | Cognitive Impairment | 0.84 | [0.74, 0.93] | <0.001*** |
|  | Emotional Impairment | 0.77 | [0.68, 0.86] | <0.001*** |
| Work Engagement | UWES Item 1 | 1.00 (ref) | — | **—** |
|  | UWES Item 2 | 1.24 | [1.10, 1.38] | <0.001*** |
|  | UWES Item 3 | 0.70 | [0.60, 0.80] | <0.001*** |

***Note.*** *λ = standardized factor loading. ref=reference, ***p < 0.001.*
